# Mitogenome Diversity and Maternal Origins of Guangxi Cattle Breeds

**DOI:** 10.3390/ani10010019

**Published:** 2019-12-20

**Authors:** Xiaoting Xia, Guangyun Huang, Zihao Wang, Junli Sun, Zhuyue Wu, Ningbo Chen, Chuzhao Lei, Quratulain Hanif

**Affiliations:** 1 Guangxi Key Laboratory of Livestock Genetic Improvement, Animal Husbandry Research Institute of Guangxi Zhuang Autonomous Region, Nanning 530001, China; xiaxiaoting1991@163.com (X.X.); hgy699@163.com (G.H.); wangzh761006@163.com (Z.W.); sjn313@126.com (J.S.); 2 Key Laboratory of Animal Genetics, Breeding and Reproduction of Shaanxi Province, College of Animal Science and Technology, Northwest A&F University, Yangling 712100, China; ningboch@126.com (N.C.); leichuzhao1118@126.com (C.L.); 3 National Institute for Biotechnology and Genetic Engineering, Pakistan Institute of Engineering and Applied Sciences, Faisalabad 577, Pakistan; micro32uvas@gmail.com

**Keywords:** mtDNA, genetic diversity, phylogeny, maternal origin

## Abstract

**Simple Summary:**

Mitochondrial DNA (mtDNA) analysis is a critical tool in assessing the maternal origin, phylogeny, and population structure of domestic animals. Guangxi cattle are located in southernmost China, where the pure Chinese indicine resources might be preserved. In this study, we sequenced the complete mtDNA of three cattle breeds in Guangxi Province for phylogenetic analysis. The aim of this study was to determine the maternal origin and phylogenetic status of Guangxi cattle.

**Abstract:**

Guangxi Province is located in the southernmost region of China, adjacent to the hotspot that is considered to be the putative migratory corridor or domestication area for Chinese indicine cattle. Here, we investigated the evolutionary status of Guangxi native breeds, Longlin (*n* = 21), Nandan (*n* = 18), and Weizhou cattle (*n* = 17) using mitogenome sequencing. Our results show that *Bos indicus* sub-haplogroup I1a predominates in Guangxi cattle breeds. Population structure by multidimensional-scaling analysis significantly differentiates Weizhou from the other two breeds (Longlin and Nandan). Moreover, the mtDNA haplotype composition and *F*_ST_ values indicate that the formation of Longlin and Nandan breeds may have been affected by Indian indicine, whereas, Weizhou island might have preserved pure Chinese indicine cattle due to its geographical isolation. We speculate that following the initial entry of zebu into southern China, the subsequent introgression of Indian indicine may have influenced the matrilineal origin of local breeds in southwestern China.

## 1. Introduction

The formation of modern Chinese cattle breeds is the result of multiple and successive invasions throughout history. Based on the morphological and genetic evidence, Chinese cattle have been mainly classified into two types: *Bos taurus* (taurine) and *Bos indicus* (indicine) [1,2,3]. *Bos taurus* originated in Near East, and then spread into East Asia and thought to have reached China at least ~3900 years before present (YBP) [4]. Previous studies indicated that *Bos indicus* originated from Indus Valley about 80000 YBP, and spread eastwards to Southeast Asia and southern China <4000 YBP [5,6]. A recent study has shown that southern China might be another domestication center for indicine cattle [4]. Indicine cattle were found to be dominant in southern China [2,3,7]. Guangxi Province represents the southernmost region in China where cattle farming has been traditionally practiced. There are three prominent cattle breeds in Guangxi Province: Nandan, Longlin, and Weizhou cattle. Nandan and Longlin cattle inhabit the northwest of Guangxi, while Weizhou cattle were introduced to Weizhou Island from the Leizhou Peninsula more than 100 years ago [8]. Leizhou Peninsula was considered to be the harbor of the pure Chinese indicine cattle [9].

Maternally inherited mitochondrial DNA (mtDNA) has been extensively used to determine the genetic variation and phylogenetic relationships of domestic cattle, but related studies have been mostly restricted to the short hypervariable region, which makes it impossible to clearly distinguish between some important ancient branches within the tree [2,5,10]. Previous studies of mtDNA described seven highly divergent haplogroups in domestic cattle: T1, T2, T3, T4, T5, I1, I2 [10,11,12]. Recently, within the *Bos indicus* lineage, a novel sub-haplogroup, named I1a, has been described by Chen et al. [4] on the basis of mitogenome haplotypes. Subsequent study based on mitogenome also confirmed the dominant position of this sub-haplogroup in Chinese local breeds [13].

Several previous studies have examined the genetic diversity and origin of Guangxi cattle breeds using both mitochondrial and Y-chromosome data. Early studies using mtDNA control region have suggested that both Nandan and Longlin have *Bos indicus* and *Bos taurus* mtDNA haplotypes while Weizhou only harbor *Bos indicus* haplotypes [13,14,15]. Another study based on the polymorphism of Y-chromosome (Y-SNP and Y-STR) showed that all the three Guangxi breeds belonged to the *Bos indicus* Y3 lineage [16]. Here, we sequenced and assembled the complete mitochondrial genomes of the three local cattle breeds in Guangxi Province, and along with seven published Indian cattle data for phylogenetic analysis. The purpose of this study was to determine the maternal origins and phylogenetic status of the southernmost cattle in China.

## 2. Materials and Methods

### 2.1. Animal Sampling and Ethics Statement

**Figure 1:**
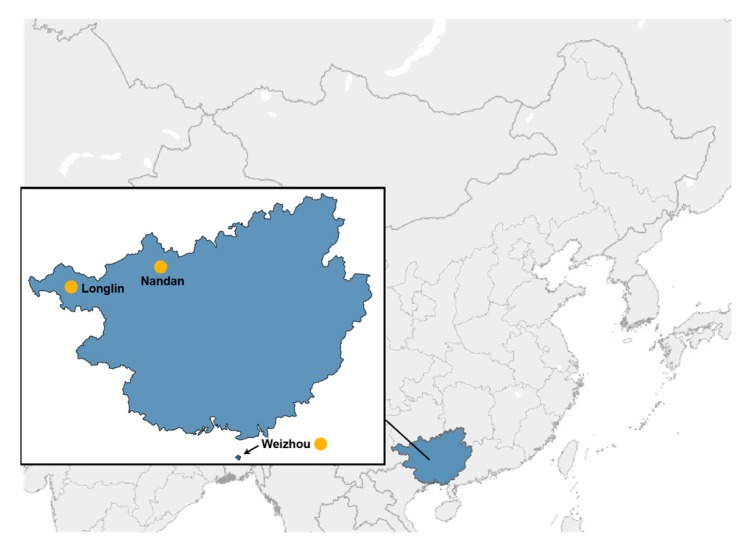
Geographical location of the sampling site.

A total of 56 ear tissues were collected from three cattle breeds, including 18 Nandan (ND), 21 Longlin (LL), and 17 Weizhou (WZ) from Guangxi Province (Figure 1, Table S1). To minimize the degree of relationship among individuals, animals were selected according to pedigree information. The protocols used in this study and for the animals were recognized by the Faculty of Animal Policy and Welfare Committee of Northwest A&F University (FAPWC-NWAFU, Protocol number, NWAFAC1008).

### 2.2. Illumina Sequencing and Reconstruction of Mitochondrial Genomes

Genomic DNA was extracted by the standard phenol-chloroform method [17]. Paired-end libraries with an average insert size of 500 bp were constructed for each individual and sequenced using the HiSeq 2000 platform (Illumina). All reads were aligned to the Bos indicus mitochondrial reference genome (NCBI: NC_005971) using the Burrows–Wheeler Aligner BWA-MEM (v0.7.13-r1126) with default options. The average depth-of-coverage was 3765.75 X ranging from 683.20 to 24,551.627 X (Table S1). BAM alignments were transformed to FASTQ files and then Mapping Iterative Assembler v 1.0 (MIA) was used to assemble a mtDNA consensus sequence.

### 2.3. Data Analysis

The complete mitochondrial genome sequence was obtained for each of the three Guangxi cattle breeds including Nandan, Longlin, and Weizhou. All the sequences were deposited in GenBank under the accession numbers MN714163–MN714218.

Measures of mitogenome sequence variation, including numbers of haplotypes and variable sites, haplotype diversity (Hd), nucleotide diversity (Pi), and the average number of nucleotide differences (k), were calculated using the program DnaSP v 5.10 [18]. The analysis of molecular variation was computed using the AMOVA program implemented in the ARLEQUIN 3.01 package [19]. A multidimensional scaling (MDS) analysis was then performed based on the matrix of *F*_ST_ values using the SPSS version 18.0 software package (SPSS, Inc. Chicago, IL, USA). A neighbor-joining tree was constructed in MEGA 5.0 [20], and the reliability of the tree topology was assessed by 1000 bootstrap replications. The median-joining network was constructed using NETWORK 5.0.1.1 [21]. The Bayesian phylogenetic tree was constructed using BEAST v2.4.5 [22] under the following parameters: HKY substitution model with eight gamma categories; lognormal strict clock model; and 20,000,000 generations sampling every 1000 generations. The remaining settings were left as defaults. Nucleotide substitution and site heterogeneity models were estimated in jModelTest v.2.1.4 [23]. Convergence was confirmed by effective sampling size (ESS) greater than 200 using the program Tracer v.1.7. We then used TreeAnnotator to summarize the MCMC samples as the maximum clade credibility topology.

## 3. Results

### 3.1. MtDNA Sequence Variation and Genetic Diversity

Analysis of mitogenome sequences (16,338 to 16,341 bp) revealed 308 variable sites among the 56 samples, defining a total of 34 haplotypes (Figure 1a). The mtDNA polymorphic sites for the three Guangxi cattle breeds are listed in Table S1. Longlin shared the highest number of haplotypes (15), followed by Weizhou (12) and Nandan (10) cattle. H34 was the most common haplotype, and only this haplotype was shared among Longlin, Nandan, and Weizhou cattle. Twenty-six haplotypes were observed only once. Estimates of mtDNA genetic diversity and haplogroup frequencies within Guangxi cattle breeds are shown in Table 1. Among them, the greatest diversity was observed in Longlin (Hd ± SD = 0.957 ± 0.030) and Weizhou (Hd ± SD = 0.949 ± 0.037) whereas the breed Nandan had the lowest estimates (Hd ± SD = 0.876 ± 0.063). The average number of pairwise differences (k) for Nandan cattle was higher than that for Longlin and Weizhou.

### 3.2. Population Phylogenetic Analysis

**Figure 2:**
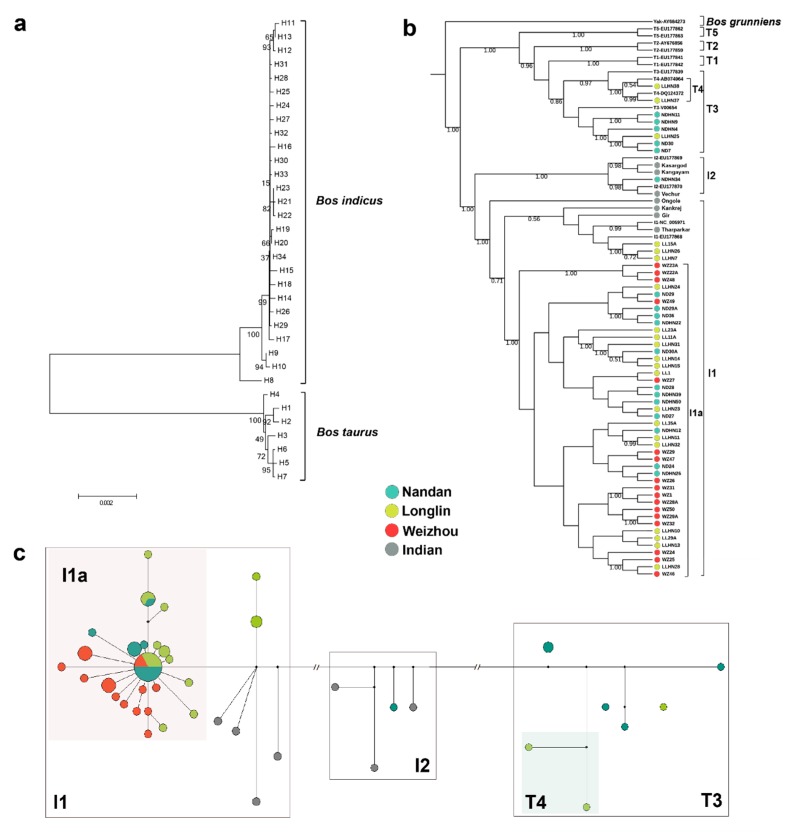
(**a**) A neighbor-joining (NJ) tree of 34 haplotypes in Weizhou, Longlin, and Nandan cattle. The values on the branches were bootstrap support based on 1000 replications. (**b**) A Bayesian phylogenetic tree of 56 Guangxi and seven Indian sequences, as well as 15 cattle reference sequences. This tree was rooted by using a *Bos grunniens* mitochondrial genome (accession no. AY684273). The values on the branches represent the posterior probabilities. The GenBank accession numbers for the haplogroup references are shown in labels. (**c**) Median-joining network constructed from 63 sequences. The areas of the circles are proportional to haplotype frequencies. Black points are median vectors.

A Bayesian phylogenetic tree was constructed based on 63 complete mitogenomes (56 Guangxi cattle in this study and seven Indian cattle from Pramod et al. [24]), combined with 14 representative sequences (T1–T5, I1, and I2) retrieved from GenBank together with *Bos grunniens* mtDNA sequence (accession no. AY684273) as an outgroup (Figure 2b). The Bayesian tree divided all Guangxi cattle into four distinct mtDNA lineages I1, I2, T3, and T4, with 26, 1, 5, and 2 haplotypes, respectively. Lineage I1 had a total of 51 individuals with Chinese (Guangxi) and Indian cattle contributing 47 (47/56, 83.93%) and four (4/7, 57.14%) individuals, respectively, while I2 had four individuals, one from Nandan (1/56, 1.79%) and three from Indian (3/7, 42.86%).

Within I1 lineage, Indian cattle clustered at the root segment, which represents the ancestral indicine mtDNA haplotypes (Figure 2b). The major feature of phylogenetic analyses in Guangxi cattle is the existence of the specific sub-lineage I1a [4]. The topology of the median-joining network reveals analogous phylogenetic relationship among the four cattle populations. The star-like pattern of this sub-haplogroup is typical for population expansions [11], presumably associated with the domestication process itself (Figure 2c).

### 3.3. Population Genetic Structure

**Figure 3:**
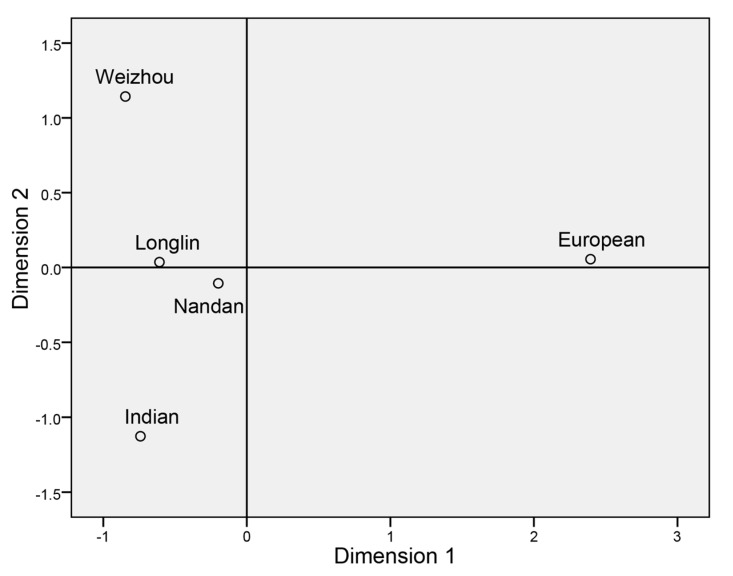
Genetic relationships between populations based on multidimensional scaling (MDS) and a matrix of the pairwise *F*_ST_ genetic distances.

The complete mtDNA genome was analyzed along with seven published mitochondrial genomes from Indian cattle to further study the matrilineal genetic structure of Guangxi cattle breeds. The computational statistical analysis revealed that most of the genetic variance was attributable within breeds (86.69%, *p* < 0.05). Geographic distribution of breeds (among groups) represented no significant effect and accounted for only 3.64% of the total variability. Estimate of pairwise-population *F*_ST_ values indicated a high genetic differentiation between Indian and Weizhou cattle (0.5231, *p* = 0.000). Among the Guangxi breeds, Weizhou was the most differentiated from the other two breeds, with *F*_ST_ values ranging from 0.221 (Nandan, *p* = 0.081) to 0.081 (Longlin, *p* = 0.081). In contrast, there was limited genetic differentiation between Nandan and Longlin (0.005, *p* = 0.324). In other words, Nandan and Longlin breeds shared a similar genetic background. MDS analysis was also performed to better visualize the breed genetic relationships using European taurine cattle (AY676855–AY676873) as an outgroup (Figure 3). As expected, the European taurine cattle appears fairly separated from all the indicine breeds, evidencing the sharp dichotomy between the mitochondrial pools of *Bos taurus* and *Bos indicus*. As shown in MDS, Longlin and Nandan cattle seemed to be influenced by both Chinese indicine and Indian indicine.

## 4. Discussion

Most published studies of the genetic diversity and phylogenetic relationships among the cattle mtDNA haplogroup lineages have been performed using the D-loop region [2,5,15]. Guangxi cattle are distributed in the major hotspots for potential Chinese indicine domestication centers [2,7,9], and little information is available about their complete mtDNA genome diversity. The present study constitutes the first assessment of the genetic diversity and population structure of cattle breeds from the southernmost China and provides the initial insights into the phylogenetic patterns of these cattle breeds.

The results of our phylogenetic analysis revealed four distinct maternal haplogroups (I1, I2, T3, and T4) in Guangxi native cattle. I1 was the predominant mtDNA haplogroup, whereas the haplogroup I2 was rare and T3 and T4 only detected in limited Longlin and Nandan cattle. The composition of the mtDNA haplogroups in Guangxi breeds were congruent with those previously presented [13,15]. Moreover, the haplotype H34 was shared by Weizhou, Longlin, and Nandan, which points towards a common ancestral population for the three breeds. It is apparent from our data that mtDNA haplotypes between Chinese and Indian cattle is quite different and there is no shared haplotype (Figure 2b). Phylogenetic analysis indicated the ancestral status of mitochondrial lineages in Indian cattle (Figure 2b). This study suggests that I1a is a unique and dominant sub-haplogroup in Guangxi and is absent in India. A similar trend involving the dominant status of I1a haplotypes has also been reported for Yunnan local cattle [25]. Therefore, our results also support the conclusion that I1a is a unique sub-haplogroup of Chinese indicine [4]. A possible explanation for this finding is that indicine cattle experienced a strong population expansion after their arrival to southern China from Indus Valley [4]. However, we cannot exclude with complete certainty a domestication of the Chinese domestic indicine group elsewhere.

The analyses of the genetic divergence reveal that Nandan cattle are much closer to Indian cattle (0.14143) than to Weizhou cattle (0.22131). In addition, three Longlin (LL15A, LLHN26, and LLHN7; haplogroup I1) and one Nandan cattle (NDHN34; haplogroup I2) carried mtDNA haplotypes closely related to Indian indicine (Figure 2b). This finding indicates the influence of Indian cattle on southern Chinese cattle in the matrilineal origin. Previous studies based on the analysis of mtDNA D-loop region also suggested a secondary introgression by Indian indicine through Yunnan-Guizhou plateau [2,14]. Actually, whole-genome-resequencing studies have revealed that cattle from Yunnan, adjacent to Guangxi, were composed of crosses with Indian-Chinese indicine genotypes [4].

All Weizhou cattle belonged to the I1a sub-haplogroup. Weizhou is an island where cattle were imported from Leizhou Peninsula 100 years ago [8]. The highest genetic differentiation observed between Weizhou and Indian cattle indicates that Weizhou cattle might represent the pure Chinese indicine ancestry due to geographical barriers. Our finding indicates that two migration phases might have occurred in the history of Chinese indicine from the perspective of maternal origin. The earliest indicine cattle population (Chinese indicine) might have been introduced <4000 YBP [5], whereas a subsequent introgression (Indian indicine) resulted from the second migration event.

## 5. Conclusions

In conclusion, our findings revealed the large genetic divergence between Weizhou and the other two Guangxi Breeds (Longlin and Nandan). Sub-haplogroup I1a was the major domestication event in southern China. Weizhou Island might preserve the precious genetic resource of Chinese indicine.

## Figures and Tables

**Table 1:** Genetic structure and diversity of Guangxi cattle breeds.

Breed	N	S	H	Haplogroup				Hd ± SD	π ± SD	k
				I1	I2	T3	T4			
Longlin	21	272	15	9	1	5		0.957 ± 0.030	0.00409 ± 0.00163	66.7524
Nandan	18	267	10	12		1	2	0.876 ± 0.063	0.00646 ± 0.00143	105.5229
Weizhou	17	22	12	12				0.949 ± 0.037	0.00024 ± 0.00003	3.8529
Total	56	308	34	33	1	6	2	0.947 ± 0.021	0.00394 ± 0.00098	64.2792

N, sample size; S, number of variable sites; H, number of haplotypes; k, the average number of differences; Hd, haplotype diversity; Pi, nucleotide diversity; SD, standard deviation.

## Supplementary Materials

The following are available online at https://www.mdpi.com/2076-2615/10/1/19/s1, Table S1: Overview of sample information and sequencing statistics, Table S2: Polymorphic sites for the mitochondrial genome haplotypes in Weizhou, Longlin, and Nandan cattle.
